# Multiple levels of linguistic and paralinguistic features contribute to voice recognition

**DOI:** 10.1038/srep11475

**Published:** 2015-06-19

**Authors:** Jean Mary Zarate, Xing Tian, Kevin J. P. Woods, David Poeppel

**Affiliations:** 1 Department of Psychology, New York University.; 2 New York University Shanghai.; 3 NYU-ECNU Institute of Brain and Cognitive Science at NYU Shanghai.; 4 Department of Neuroscience, Max Planck Institute (MPIEA).

## Abstract

Voice or speaker recognition is critical in a wide variety of social contexts. In this study, we investigated the contributions of acoustic, phonological, lexical, and semantic information toward voice recognition. Native English speaking participants were trained to recognize five speakers in five conditions: non-speech, Mandarin, German, pseudo-English, and English. We showed that voice recognition significantly improved as more information became available, from purely acoustic features in non-speech to additional phonological information varying in familiarity. Moreover, we found that the recognition performance is transferable between training and testing in phonologically familiar conditions (German, pseudo-English, and English), but not in unfamiliar (Mandarin) or non-speech conditions. These results provide evidence suggesting that bottom-up acoustic analysis and top-down influence from phonological processing collaboratively govern voice recognition.

Voice recognition, irrespective of the speech content, is crucial in many social contexts, including distinguishing voices of one’s kin from those of strangers. The social relevance of voice recognition is reinforced by evidence of fetal recognition of mothers’ voices *in utero*^1^ and increasing specialization of neural mechanisms for human voices over the first six months of development^2^,^3^. These early voice-recognition mechanisms precede fully developed linguistic abilities^4^ and may therefore rely principally on acoustic, paralinguistic characteristics of voice [e.g., average fundamental frequency (F0), F0 contour, etc.] that can exist outside of the speech domain^5^.

Voice timbre — the Gestalt of sound characteristics that make a voice unique and recognizable — is determined by the physical characteristics of the vocal folds that affect speaking fundamental frequency (F0), the vocal tract, and the articulators that modify the shape of the vocal tract and influence the higher harmonics or formant frequencies of the voice, i.e., lips, teeth, jaw, tongue, etc.;^6^. Average speaking F0 (a key voice characteristic), higher-order characteristics of F0 contour, and vocalization rate or speed are important for voice recognition when phonological information is held constant and lexical semantic information is not available^7^. With reversed speech, which eliminates lexical and semantic information and distorts temporally based, consonant-related phonological information, listeners can still use voice timbre conveyed in the formant frequencies of vowels to distinguish between and recognize voices^8^,^9^. Compared to sine-wave speech — which possesses a complex timbre with phonological features, but is ultimately devoid of vocal F0 — the average speaking F0, F0 contour, and natural voice timbre in normal and reversed speech greatly enhanced listeners’ ability to distinguish between voices^8^,^10^.

Besides the paralinguistic factors that contribute to voice recognition, in a recent paper Perrachione *et al.*^11^ suggest that voice recognition depends on the integrity of the phonological representations of words. They argue that the unfamiliar phonology of a foreign language or, alternatively, a pre-existing deficit in phonological processing such as that described in dyslexia^12^ can reduce voice recognition; this implies that intact phonological processing is critical to correctly identifying a speaker^11^,^13^.

Such evidence suggests that both linguistic and paralinguistic characteristics underlie voice recognition, although this is based mostly on studies that isolate only one class of characteristics. To the best of our knowledge, no direct evidence from a single experiment has demonstrated the relative contributions of both types of characteristics towards voice recognition. Moreover, studies reporting that “language familiarity” affects voice recognition do not clearly define and operationalize this concept; language familiarity should include at least phonological familiarity (which has a number of attributes, including phonetic and stress-class similarities) as well as potential lexical and semantic access. Here, in an auditory psychophysical experiment, we examined the contributions of linguistic and paralinguistic components towards recognizing speakers by testing a hierarchical scheme — from acoustic to phonological to lexical and semantic information — and qualitatively determining what type of information modulates voice recognition.

In an experimental design modeled closely on Perrachione’s (2007, 2011) studies of voice recognition, monolingual English speakers were trained to associate five voices with avatars in five conditions (non-speech, Mandarin, German, pseudo-English, and English) — rather than just two conditions as in previous work (Mandarin and English). Three factors distinguish this from Perrachione’s earlier design. First, whereas previously speakers were changed between conditions, we used the *same speakers* and avatar-voice pairings across conditions to keep the voice sources constant. The use of the same speakers across different languages – i.e., highly competent and fluent multi-lingual speakers – is a relevant experimental choice to better distinguish the contributions of paralinguistic (e.g., acoustic) from linguistic (e.g., phonological, lexical, and semantic) cues to voice recognition. Second, we employed strings of single words or sounds to control the amount of auditory information presented in each trial – four tokens per trial here versus full sentences of varying lengths in Perrachione *et al.*’s experiments. While it can be argued that word strings (as opposed to sentences) reduce prosody that may help participants distinguish between languages, we utilized word strings for all speech conditions specifically to isolate the features of interest – acoustic, phonological, lexical, and semantic cues – rather than focusing on additional cues, such as prosody (at the intonation contour level). Third, besides the difference in voice recognition performance, the contribution of different types of information could also be reflected in extent of generalization of voice recognition from training to testing stimuli sets. While the previous work could have introduced performance bias because of partial overlaps between stimuli used in training and testing, the stimuli used for training completely differed from those used for testing in our study, such that we could examine generalization of voice recognition performance in a more unbiased fashion and assess the contributions of learned acoustic, phonological, lexical, and semantic cues towards recognizing voices in new stimuli.

We outline here a set of predictions. Based on the view that voice recognition relies only on paralinguistic or acoustic characteristics, we predicted that recognition performance would be equal across types of cues. On the other hand, if each type of information has a distinctive contribution, voice recognition performance should improve systematically as a function of the amount of information available: from acoustic features (non-speech) to the availability of unknown/unfamiliar phonological information (Mandarin) to increasingly familiar phonological content (from German to pseudo-English) to full lexical and semantic access (English). In other words, if availability of phonological information is a factor that affects voice recognition, the addition of unfamiliar Mandarin phonology to acoustic information should boost performance relative to the non-speech condition. If phonological familiarity is another factor that can influence voice recognition, performance with Mandarin should be worse compared to German, which has similar – and arguably more familiar – phonology to the participants’ native English^14^,^15^. Since the German, pseudo-English, and English conditions have overlapping phonological information, perhaps the principal distinctions between these conditions are lexical-semantic access. If lexical-semantic access further contributes to voice recognition in the current experimental context, we might see increased voice recognition progressing from German to pseudo-English, and finally to English; otherwise, we expected to see similar performance among these three conditions.

## Methods

### Participants

Eighteen right-handed participants were recruited from the New York University (NYU) community and surrounding areas. Data from two participants were excluded (see “Data exclusions” below). The remaining 16 participants (eight female; mean ± SD age = 23.3 ± 6.9 years) were monolingual English speakers, had normal hearing and had no neurological disorders. No participant was familiar with German or Mandarin or the names or voices of our speakers. All testing occurred with the participants’ informed written consent, in accordance with procedures approved by the NYU University Committee on Activities Involving Human Subjects.

### Stimuli

All vocal sounds were recorded (44100 Hz, 16-bit, stereo .wav files, normalized for amplitude) from five male speakers (fluently bilingual in English and German) who were trained and coached to generate the other conditions’ sounds. In the English conditions, two of the speakers had German accents, while the other three had an American accent; in contrast, all five speakers used German accents when producing German tokens.

Importantly, a male native Mandarin speaker coached our speakers during Mandarin-token production. Although (presumably) these speakers automatically imposed some of the phonetic properties of their native language while producing Mandarin tokens, if there were differences in voice recognition performance between Mandarin and the other conditions, we would interpret this as supporting evidence that the recorded Mandarin tokens provided enough distinct phonological content relative to tokens in other languages.

**Figure 1:**
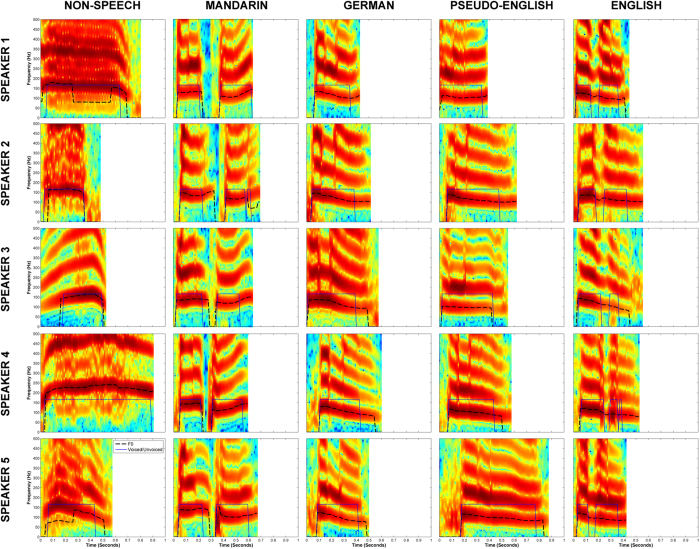
Spectral characteristics of example tokens from all five speakers (rows) and all conditions (columns): grunts (non-speech); Mandarin translation of “heaven” 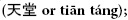 German translation of “heaven” (“himmel”); pseudo-English token “henai” constructed from “heaven” and “deny”; English word “heaven”. Black dashed lines indicate the fundamental frequency (F0, or perceived pitch) within each token; blue-outlined plateaus (arbitrary units) show the portions of each token that contained a voice timbre (“voiced”).

All speakers produced each item from the entire corpus of materials, which consisted of fifty tokens divided among five conditions (see Supplementary Information). For the *non-speech* condition, we selected 10 vocal sounds with as little phonetic content as possible (e.g., laugh, cough, cry, grunt). For the *English* condition, we selected 10 bisyllabic words from the MRC Psycholinguistic Database Machine Usable Dictionary, version 2.00^16^;, with Kucera-Francis^17^ frequency values ranging from 10–63 (out of approximately one million words). To hold the phonological information relatively constant between English words and pseudowords for the *pseudo-English* condition, we decomposed the words into their constituent syllables and created new syllable pairs to form the pseudowords. The English words were translated into *German* and *Mandarin*, resulting in 10 tokens for each condition with similar lexical and semantic content (see Appendix). Only one German token was a stand-alone word not translated from the English word “liquor” because there was no bisyllabic German equivalent; instead, we picked a bisyllabic German word (“stapfend”) comprised of phonemes that would make the German and English/pseudo-English conditions more phonologically similar. Figure 1 displays the spectral characteristics of one-tenth of our stimuli, across all five speakers and five conditions; spectrograms were generated with the YAAPT Pitch Tracking MATLAB function^18^.

Within each condition, four of the 10 tokens were chosen at random and presented solely as training stimuli. The remaining six tokens were used as test stimuli, presented in groups of four for each test trial; this was done to prevent testing-performance bias stemming from familiarity with training stimuli (see Perrachione *et al.*, 2007; 2011). In all trials, tokens were concatenated in a pseudo-randomized order with 500-ms inter-token intervals of silence (presentation length range: 3.0–5.3 s).

### Experimental procedure

Participants sat at a computer, fitted with headphones (Sennheiser HD 280 Professional, Sennheiser Electronic Corporation, Wedemark, Germany). All stimuli were delivered at a comfortable loudness level (~72 dB SPL A). The experiment was controlled using Psychtoolbox in MATLAB (MathWorks, Inc., Natick, MA, USA). The five voice-discrimination conditions (English, pseudo-English, German, Mandarin, non-speech) were presented separately in blocks comprised of 10 training and 60 test trials; block orders were pseudo-randomized across participants.

**Figure 2:**
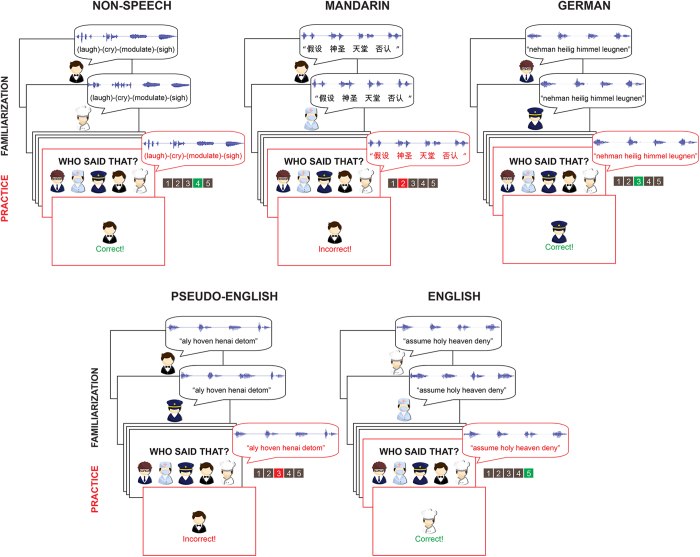
A training trial in each condition. During familiarization (black-outlined screens), one avatar is presented simultaneously with a voice producing four tokens. After all five avatar-voice pair presentations, a practice trial begins (red-outlined screens), in which a voice produces the same four tokens, and then all five avatars are shown for the voice-recognition task. Feedback is provided after their responses. The avatars used in this figure were drawn by us using Adobe^®^ Flash^®^.

In *training trials*, participants were presented with a token from all five speakers, each paired with a cartoon avatar; all five speakers were presented in every trial. These pairings were picked randomly and were maintained across the entire experiment for each participant. After all five avatar-voice pairings were presented, the voice from a randomly chosen speaker was played again. Participants indicated the correct speaker among the five avatars shown on the screen (using keyboard buttons 1–5) in speeded trials; reaction times and accuracy were measured, and feedback was provided. After 10 training trials, participants performed 60 *test trials*, during which four new tokens (out of six possible non-trained tokens) were presented from only one speaker in each trial. Participants selected the correct avatar for that speaker, and no feedback was provided. Figure 2 illustrates trial structure.

### Analyses

We performed one-way repeated-measures analyses of variance (RM-ANOVAs, with condition as the within-subject factor) on the percent-correct scores and reaction times. Bonferroni-corrected *t*-tests were used for both post-hoc tests of significant main effects and planned comparisons in significant interactions. Correlation analyses were performed to assess effects of condition-block order on performance.

To evaluate the transfer of learned avatar-voice pairings from training to testing (with different stimuli and no feedback), we divided the data into seven 10-trial bins: the first bin for training, and the subsequent 6 bins for testing. We used two-way RM-ANOVAs (condition x time bin) to analyze performance and reaction times in each condition, and performed planned comparisons between the first two bins — i.e., the 10 training trials versus the first 10 test trials — to assess learning transfer from training to test phases. We also analyzed the slopes calculated across percent-correct scores and reaction times from the six test-phase bins in each condition to evaluate procedural learning (i.e., significant changes in percent-correct scores and/or in reaction times) during the testing phase.

### Data exclusions

Of the 18 enrolled participants, we excluded the data from one participant due to experimental error (MATLAB scripting error) and from another due to task performance; a Dixon Test^19^ for rejection of outliers determined that this participant’s performance was statistically different from all others.

We excluded reaction times that were outside three standard deviations from the mean reaction time calculated from 350 trials [5 conditions x 70 trials (10 training, 60 testing)] for each participant. For each participant, 3.14 ± 0.34 (mean ± S.E.M.) trials were excluded.

## Results

### Voice recognition: condition and block-order effects

**Figure 3:**
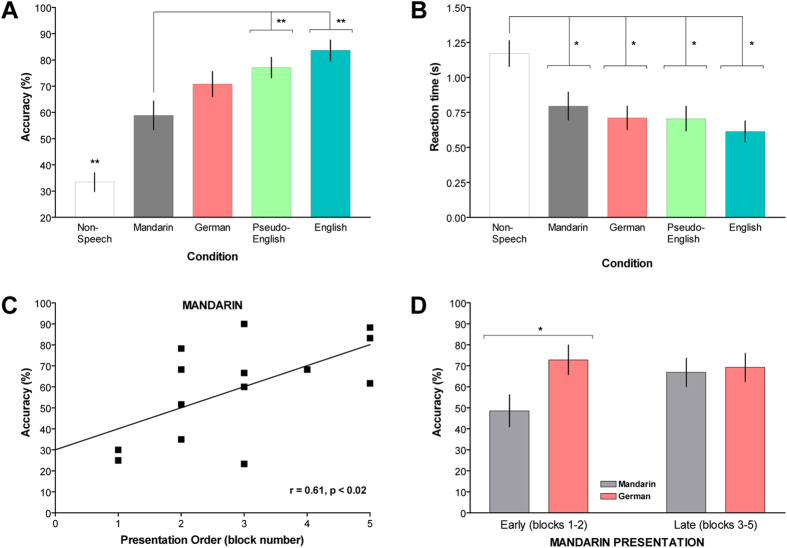
Average performance measures in voice recognition. **A**) Voice recognition with non-speech was less accurate than all speech conditions, and accuracy in Mandarin was significantly lower than in pseudo-English and English (** denotes *p *< 0.01). **B**) Reaction times during the non-speech condition were longer than during speech conditions (* indicates *p *< 0.05). **C**) Voice recognition significantly correlated with the order of experimental presentation only in Mandarin, such that performance improved as it was presented later within the experiment. **D**) Compared to performance in German, voice recognition in Mandarin was worse when it was presented early in the experiment (*p *< 0.05), whereas performance between the two languages were similar in later blocks.

Figure 3A shows that there is improvement in voice recognition as more information becomes available. Participants performed at 33.4% accuracy with non-speech (chance is 20%). The introduction of phonological information improves performance almost two-fold (58.9% with Mandarin, an unfamiliar language) and significantly higher with phonologically familiar languages. A one-way RM-ANOVA was performed on the percent-correct scores from all participants, with condition as the within-subject factor. We found a significant effect of condition [Fig. 3A; *F*(4,60) = 31.79, *p *< 0.001]. The linear and quadratic components of the RM-ANOVA were statistically significant [linear: *F*(1,15) = 136.16, *p *< 0.001; quadratic: *F*(1,15) = 8.28, *p *< 0.05]. The significance of the quadratic component is due presumably to the significant increase in performance between non-speech and all language conditions. Indeed, post-hoc comparisons determined that performance was lower for the non-speech condition than for Mandarin, German, pseudo-English, and English (Fig. 3A, *p*s < 0.01). Performance in Mandarin was worse than performance in both pseudo-English and English (*p*s < 0.01), but we found no differences between Mandarin and German (*p *> 0.4). We also found no statistical differences in performance between German, pseudo-English, and English (*p*s > 0.2). A one-way RM-ANOVA on reaction times resulted in a significant condition effect [F(4,60) = 13.89, *p *< 0.001]. Post-hoc comparisons revealed that the average reaction time during non-speech was longer than reaction times from all other conditions (Fig. 3B; *p*s < 0.05).

To determine if condition-block order may have influenced recognition accuracy and resulted in the lack of significant differences between Mandarin and German and between German, pseudo-English, and English, we conducted correlation analyses between condition-block order and accuracy scores in each condition. Voice recognition in the Mandarin condition improved when it was presented later in the experiment [Fig. 3C; *r*(14) = 0.61, *p *< 0.02]; all other correlations between condition-block order and accuracy were not significant (*p*s > 0.2). This order effect may be responsible for the lack of differences in voice recognition between Mandarin and German. Consequently, we grouped the participants based on when Mandarin was presented: an early group (in either of the first two blocks) and a late group (in the third, fourth, or last test block). We performed a two-way mixed RM-ANOVA (group-by-condition) on the percent-correct scores in Mandarin and German, which resulted in a significant main effect of condition [*F*(1,14) = 7.22, *p *< 0.05] and a significant group-by-condition interaction [Fig. 3D; *F*(1,14) = 4.85, *p *< 0.05]. Planned comparisons determined that participants who were tested earlier with Mandarin had worse voice recognition relative to German (*p *< 0.05), while participants who were tested later with Mandarin performed similarly as in German (*p *> 0.6).

To more directly test our prediction that increasing phonological familiarity would improve voice recognition, we ran a one-way RM-ANOVA including only the Mandarin, German and pseudo-English conditions in which the phonological familiarity was manipulated. We found a significant effect of condition on voice recognition accuracy [*F*(2,30) = 8.33, *p *< 0.01]. More importantly, the linear component of the RM-ANOVA was statistically significant [*F*(1,15) = 31.45, *p *< 0.001], which suggests that voice recognition improved as a function of increasingly familiar phonological familiarity.

### Voice recognition: from training to testing

**Figure 4:**
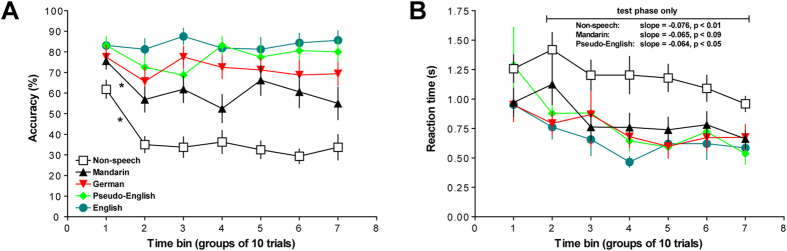
Voice-recognition performance across training and testing. **A**) Voice recognition decreased from training (time bin 1) to the beginning of testing (time bin 2) only in the non-speech and Mandarin conditions (* equals *p *< 0.05). **B**) Reaction times decreased across testing in only non-speech, Mandarin, and pseudo-English (*p*s < 0.09).

Figure 4A shows that voice-recognition accuracy during training (time bin 1) is maintained in testing with novel stimuli (time bins 2–7) in phonologically familiar conditions (German, pseudo-English, English), while performance worsens after introducing novel non-speech and Mandarin test stimuli. Figure 4B demonstrates that these changes in accuracy (or lack thereof) are not associated with significant changes in reaction time. A two-way RM-ANOVA (condition x time bin) performed on the percent-correct scores (from successive time bins of 10 trials from training and test phases; see Methods for details) resulted in significant main effects of condition [*F*(4,60) = 31.64, *p *< 0.001], time bin [*F*(6,90) = 6.30, *p *< 0.001] and a significant condition-time bin interaction [Fig. 4A; *F*(24,360) = 1.97, *p *< 0.01]. Planned comparisons between the first two time bins (i.e., 10 training trials in time bin 1 versus the first 10 test trials in time bin 2) determined that voice recognition was worse during the first 10 test trials (compared to training) in the non-speech and Mandarin conditions only (Fig. 4A; *ps *< 0.05); there were no significant differences in performance between training and test phases in the other three conditions (*p*s > 0.2). In other words, the learned associations between voices and avatars were more successfully transferred between training and test phases of German, pseudo-English, and English than during non-speech and Mandarin. None of the slopes calculated across the six test-phase bins were significantly different from zero, indicating that recognition accuracy did not significantly change during the test phases of any condition (*p*s > 0.1).

The two-way RM-ANOVA performed on the reaction times from all testing and testing time bins (Fig. 4B) revealed significant effects of condition [*F*(4,60) = 10.46, *p *< 0.001] and time bin [*F*(6,90) = 8.69, *p *< 0.001]; no significant two-way interaction was found (*p *> 0.2). Post-hoc tests performed on each main effect determined that: 1) non-speech elicited the longest reaction times across the entire block, compared to all other conditions (*p*s < 0.01); and 2) participants took longer to answer during training and the first 10 test trials than the rest of the test phase, regardless of the condition presented (*p*s < 0.06). The slopes calculated from reaction times during non-speech, Mandarin, and pseudo-English conditions were significantly different from zero, indicating that participants got faster towards the end of these particular test phases [Fig. 4B; non-speech slope = −0.076 ± 0.014 (S.E.M.), *p *< 0.01; Mandarin slope = −0.065 ± 0.029 (S.E.M.), *p *< 0.09; pseudo-English slope = −0.064 ± 0.022 (S.E.M.), *p *< 0.05]; the other slopes were not significantly different from zero (*p*s > 0.1). Together with the slope analyses using percent-correct scores above, participants may have gotten faster during testing in a few conditions (as shown by our slope analyses in Fig. 4B), but this was not concomitant with a significant change in accuracy throughout testing. Thus, we conclude that voice recognition was relatively constant throughout the test phases once feedback was removed.

## Discussion

### Voice recognition as a function of availability of acoustic and phonological features

We trained participants to recognize five voices in five conditions: non-speech, Mandarin, German, pseudo-English, and English. Our analyses demonstrate that voice recognition improved as a function of available increasing amounts of information (e.g., average speaking F0, phonotactics, etc.). Voice recognition with non-speech (the condition with the least amount of phonological information) was lower than in all other conditions but above chance, suggesting that purely acoustic, paralinguistic features such as voice timbre and average speaking F0 (across all tokens) may be sufficient for recognizing voices, consistent with van Dommelen’s (1990) findings. Performance in Mandarin — a condition with phonetic and phonological information, albeit unfamiliar relative to German — was higher than in non-speech, demonstrating that coupling any phonetic and phonological information with acoustic information greatly enhances voice recognition.

Recognition is further enhanced when proceeding from unfamiliar (Mandarin) to more familiar phonology (German) within the first two experimental blocks. Additionally, the performance in the pseudo-English and English conditions was much better than that in Mandarin condition. These results suggest that phonological familiarity influences voice recognition. (The graded response between Mandarin and German also suggests that our Mandarin tokens from non-native speakers provided distinct phonological information and was not ‘contaminated’ by German or English phonetics, thus validating our design.) Because the processing and extraction of phonological features does not change based on individual acoustic features like fundamental frequency^20^, there is limited information available in the abstract phonological features for identifying individual voices. The information that can be used to differentiate people’s voices is mostly included in the acoustic signals. Therefore, the effect of phonological familiarity on voice recognition observed in this study is presumably via a top-down influence on basic acoustic analysis of incoming speech.

Due to an order effect in Mandarin, this difference between foreign languages disappears in the last three blocks: voice recognition improved as Mandarin was presented later within the experiment. Based on these findings and previous work e.g.,^21^,^22^, voice recognition with unfamiliar phonology (such as in Mandarin for our native English listeners) can improve with increased exposure to paralinguistic features (e.g., voice timbre, average speaking F0, etc.).

Voice recognition did not differ across condition blocks with high phonological familiarity (German, pseudo-English, and English); furthermore, within each of these condition blocks, performance did not significantly change during transfer from training stimuli to new test stimuli. These observations of robust, similar performances across the German and English conditions contradict earlier reports of reduced voice recognition in an unfamiliar language such as German^23^,^24^. It should be noted that those studies utilized excerpts of text written in or translated to German; when linguistic information in this text was reduced, such that all syllables are replaced with /ma/, language familiarity no longer influences voice recognition^25^. The lack of differences in voice recognition observed in both our study — where bisyllabic words/pseudowords were presented — and Schiller’s study suggests that lexical and semantic information are not necessary for successful voice recognition, at least in these particular experimental procedures. It can be argued that full lexical and semantic information may depend on other cues that are not available in monosyllabic stimuli or bisyllabic words. Yet in Perrachione *et al.*’s (2011) study that employed full sentences in English and Chinese, although all native-English participants had full lexical and semantic access when comparing voices in English (relative to Chinese), participants with dyslexia demonstrated no significant differences in voice recognition across the two languages. Since dyslexic participants also exhibited larger deficits in phonological processing, their performance may have suffered from an inability to recognize idiosyncratic phonetic features from each speaker^11^. Together, our results clarify the concept of “language familiarity” and its influence on voice recognition: phonological familiarity, and not lexical and semantic information, is more crucial for voice recognition.

## Conclusion

Overall, we show that multiple levels of acoustic and increasingly familiar phonological information contribute to voice recognition, expanding upon previous accounts of voice recognition. We also provide direct evidence supporting the importance of phonological — and *not* overall language — familiarity in voice recognition, after eliminating the confound of lexical-semantic access. These results provide evidence suggesting that bottom-up acoustic analysis and top-down phonological processing collaboratively govern voice recognition.

## Additional Information

**How to cite this article**: Zarate, J.M. *et al.* Multiple levels of linguistic and paralinguistic features contribute to voice recognition. *Sci. Rep.* **5**, 11475; doi: 10.1038/srep11475 (2015).

## Supplementary Material

Supplementary Information

## References

[b1] Kisilevsky B. S. *et al.* Effects of experience on fetal voice recognition. Psychological Science 14, 220–224 (2003). 12741744 10.1111/1467-9280.02435

[b2] Lloyd-Fox S., Blasi A., Mercure E., Elwell C. E. & Johnson M. H. The emergence of cerebral specialization for the human voice over the first months of life. Social Neuroscience 7, 317–330 (2012). 21950945 10.1080/17470919.2011.614696

[b3] Vouloumanos A., Hauser M. D., Werker J. F. & Martin A. The tuning of human neonates’ preference for speech. Child Dev 81, 517–527 (2010). 20438457 10.1111/j.1467-8624.2009.01412.x

[b4] Kuhl P. K. Early language acquisition: cracking the speech code. Nat Rev Neurosci 5, 831–843 (2004). 15496861 10.1038/nrn1533

[b5] Ladefoged P. & Broadbent D. E. Information conveyed by vowels. Journal of the Acoustical Society of America 29, 98–104 (1957). 10.1121/1.397821 2525139

[b6] Sundberg J. The Science of the Singing Voice. (Northern Illinois University Press, 1987).

[b7] van Dommelen W. A. Acoustic parameters in human speaker recognition. Language and Speech 33, 259–272 (1990). 2093780 10.1177/002383099003300302

[b8] Remez R. E., Fellowes J. M. & Nagel D. S. On the perception of similarity among talkers. J Acoust Soc Am 122, 3688–3696 (2007). 18247776 10.1121/1.2799903

[b9] Van Lancker D., Kreiman J. & Emmorey K. Familiar voice recognition: patterns and parameters. Part I: Recognition of backward voices. Journal of Phonetics 13, 19–38 (1985).

[b10] Vouloumanos A., Hauser M. D., Werker J. F. & Martin A. The tuning of human neonates’ preference for speech. Child development 81, 517–527 (2010). 20438457 10.1111/j.1467-8624.2009.01412.x

[b11] Perrachione T. K., Del Tufo S. N. & Gabrieli J. D. Human voice recognition depends on language ability. Science 333, 595 (2011). 21798942 10.1126/science.1207327 PMC4242590

[b12] Gabrieli J. D. Dyslexia: a new synergy between education and cognitive neuroscience. Science 325, 280–283 (2009). 19608907 10.1126/science.1171999

[b13] Perrachione T. K. & Wong P. C. Learning to recognize speakers of a non-native language: implications for the functional organization of human auditory cortex. Neuropsychologia 45, 1899–1910 (2007). 17258240 10.1016/j.neuropsychologia.2006.11.015

[b14] Müller K. Revealing phonological similarities between related languages from automatically generated parallel corpora. *Proceedings of the Association for Computational Linguistics Workshop on Building and Using Parallel Texts*, 33-40, Ann Arbor, MI. Stroudsburg, PA: Association for Computational Lingustics. (2005).

[b15] Ziegler J. C. & Goswami U. Reading acquisition, developmental dyslexia, and skilled reading across languages: a psycholinguistic grain size theory. Psychol Bull 131, 3–29 (2005). 15631549 10.1037/0033-2909.131.1.3

[b16] Coltheart M. The MRC Psycholinguistic Database. Quarterly Journal of Experimental Psychology 33A, 497–505 (1981).

[b17] Kucera H. & Francis W. N. Computational Analysis of Present-Day American English. (Brown University Press, 1967).

[b18] Zahorian S. A. & Hu H. A spectral/temporal method for robust fundamental frequency tracking. J Acoust Soc Am 123, 4559–4571 (2008). 18537404 10.1121/1.2916590

[b19] Dixon W. J. Analysis of Extreme Values. Ann Math Stat 21, 488–506 (1950).

[b20] Phillips C. *et al.* Auditory cortex accesses phonological categories: an MEG mismatch study. Journal of Cognitive Neuroscience 12, 1038–1055 (2000). 11177423 10.1162/08989290051137567

[b21] Levi S. V., Winters S. J. & Pisoni D. B. Effects of cross-language voice training on speech perception: whose familiar voices are more intelligible? J Acoust Soc Am 130, 4053–4062 (2011). 22225059 10.1121/1.3651816 PMC3253604

[b22] Winters S. J., Levi S. V. & Pisoni D. B. Identification and discrimination of bilingual talkers across languages. J Acoust Soc Am 123, 4524–4538 (2008). 18537401 10.1121/1.2913046 PMC2680657

[b23] Goggin J. P., Thompson C. P., Strube G. & Simental L. R. The role of language familiarity in voice identification. Memory & Cognition 19, 448–458 (1991). 1956306 10.3758/bf03199567

[b24] Köster O. & Schiller N. Different influences of the native language of a listener on speaker recognition. Forensic Linguistics 4, 18–28 (1997).

[b25] Schiller N., Köster O. & Duckworth M. The effect of removing linguistic information upon identifying speakers of a foreign language. Forensic Linguistics 4, 1–17 (1997).

