# Supplementary material for: Multiple levels of linguistic and paralinguistic features contribute to voice recognition
Source: Sci Rep. 2015 Jun 19;5:11475. doi: 10.1038/srep11475 (PMC4473599; doi:10.1038/srep11475)
Supplement: Supplementary Information [file srep11475-s1.doc]

Supplementary Materials

Multiple levels of linguistic and paralinguistic features contribute to voice recognition

by

Jean Mary Zarate^1,#^, Xing Tian^1,2,#^, Kevin J.P. Woods^1^, and David Poeppel^1,3^

^1^ Department of Psychology, New York University

^2^ NYU-ECNU Institute of Brain and Cognitive Science at NYU Shanghai

^3^ Department of Neuroscience, Max Planck Institute (MPIEA)

Supplementary Table 1. List of stimuli

| English words:  assume  holy  heaven  deny  atom  bacon  funny  reader  liquor  copy | German translations:  nehmen  heilig  himmel  leugnen  teilchen  schinken  komisch  leser  stapfend  zweitschrift | Mandarin translations:  假设 ([jiǎ](http://www.mandarintools.com/sounds/jia3.aif) [shè](http://www.mandarintools.com/sounds/she4.aif))  神圣 ([shén](http://www.mandarintools.com/sounds/shen2.aif) [shèng](http://www.mandarintools.com/sounds/sheng4.aif))  天堂 ([tiān](http://www.mandarintools.com/sounds/tian1.aif) [táng](http://www.mandarintools.com/sounds/tang2.aif))  否认 ([fǒu](http://www.mandarintools.com/sounds/fou3.aif) [rèn](http://www.mandarintools.com/sounds/ren4.aif))  原子 ([yuán](http://www.mandarintools.com/sounds/yuan2.aif) [zǐ](http://www.mandarintools.com/sounds/zi3.aif))  咸肉 ([xián](http://www.mandarintools.com/sounds/xian2.aif) ròu)  有趣 ([yǒu](http://www.mandarintools.com/sounds/you3.aif) [qù](http://www.mandarintools.com/sounds/qu4.aif))  读者 ([dú](http://www.mandarintools.com/sounds/du2.aif) [zhě](http://www.mandarintools.com/sounds/zhe3.aif))  烈酒 ([liè](http://www.mandarintools.com/sounds/lie4.aif) [jiǔ](http://www.mandarintools.com/sounds/jiu3.aif))  复印 ([fù](http://www.mandarintools.com/sounds/fu4.aif) [yìn](http://www.mandarintools.com/sounds/yin4.aif)) |
| --- | --- | --- |
| English pseudowords:  aly (uh-lee)  hoven (HOE-vun)  henai (heh-nai)  detom (di-tum)  ackon (Æ-konn)  bainy (BAY-nee)  fudder (FUH-der)  reeker (REE-ker)  lippy (LIH-pee)  cosume (cah-SUME) |  | Vocal, non-speech sounds:  two (2) tokens of laughing  two (2) tokens of crying  two (2) continuously modulated drones  voiced cough  voiced sigh  grunt  voiced heaving (lifting heavy object) |
